# EFSA Health Claims-Based Virgin Olive Oil Shelf-Life

**DOI:** 10.3390/antiox12081563

**Published:** 2023-08-04

**Authors:** Vanessa Mancebo-Campos, Maria Desamparados Salvador, Giuseppe Fregapane

**Affiliations:** Department of Analytical Chemistry and Food Technology, Faculty of Chemistry, University of Castilla-La Mancha, 13071 Ciudad Real, Spain; amparo.salvador@uclm.es (M.D.S.); giuseppe.fregapane@uclm.es (G.F.)

**Keywords:** virgin olive oil, shelf life, olive oil storage, accelerated shelf-life test, health claim, olive oil phenolic compounds, antioxidants, European Commission Regulation 432/2012

## Abstract

The consumption of extra virgin olive oil (EVOO) has been linked to various health benefits, including a reduced risk of cardiovascular disease. EVOO contains triglycerides and unsaturated fatty acids, as well as minor compounds, such as polar phenols and tocopherols, which play a crucial nutritional and biological role. The composition of these minor compounds is affected by various factors that distinguish EVOOs from lower-quality olive oils. The European Parliament approved Regulation 1924/2006 that governs the use of health claims on food products based on EFSA reports. Currently, there are several authorized health claims related to unsaturated fatty acids, vitamin E, and polyphenol content that can be used for commercial reasons on EVOO labels. Consumers can easily take enough grams of EVOO per day to receive the beneficial effects of the nutrient in question; nevertheless, the use of these health claims is subject to a required concentration of specific nutrients throughout the shelf life of olive oil. Few studies have examined the evolution of these compounds along the shelf life of EVOO to meet health claims. This work aims to evaluate the nutritional profile of several EVOOs with potential health claims and the evolution of related nutrients during storage in darkness at different temperatures. This study proposes an accelerated method to determine the end of the EVOO shelf life based on the loss of its nutraceutical capacity and the inability to comply with the stated health claims.

## 1. Introduction

The consumption of olive oil is one of the fundamental pillars supporting the Mediterranean diet. The beneficial effects associated with the frequent intake of olive oil have been widely described and recognized [1,2,3,4,5,6]. The PREDIMED project revealed a considerably lower risk of cardiovascular disease for individuals who consume olive oil. In fact, they recommended a preference for extra virgin olive oil (EVOO) [7,8,9] as compared to other edible fats.

The lipid oxidation of olive oil is, like most edible fats, its main cause of alteration and degradation, resulting in a reduction of its commercial quality and especially of its nutritional and biological value. Fortunately, the antioxidant activity of some of its minor components delays the progress of the oxidation process, helping to protect the added value of this food product [10,11].

The components of olive oil are divided into saponifiable (triglycerides and free fatty acids, marked by a predominance of monounsaturated FAs, especially oleic acid), covering 98.0–99.0%, and unsaponifiable (hydrocarbons, aliphatic and aromatic alcohols, phenols, sterols, tocopherols, fat-soluble vitamins, volatile organic compounds, aldehydes, triterpenic acids, etc.), which while occupying a small percentage, play an important nutritional and biological role [12]. The qualitative and quantitative composition of this minor fraction is greatly influenced by many variables related to the variety of the olive, the cultivation area, the applied cultivation techniques, the environmental–climatic conditions, the degree of ripeness of the fruit, the method of collecting the fruit, the type of processing applied during production and storage conditions [13] and differentiates EVOOs from lower-quality olive oils.

The phenolic fraction of olive oil consists of a heterogeneous mixture of compounds with the most abundant being the simple phenols hydroxytyrosol and tyrosol (3,4-DHPEA and p-HPEA); the aglycones of oleuropein and ligstroside; the aldehydic forms of oleuropein aglycon and ligstroside aglycon (3,4-DHPEA-EA and p-HPEA-EA), which belong to the secoiridoids; the dialdehydic form of decarboxymethyl elenolic acid linked to hydroxytyrosol (oleacein, 3,4-DHPEA-EDA); the dialdehydic form of decarboxymethyl elenolic acid linked to tyrosol (oleocanthal, p-HPEA-EDA); lignans (1-acetoxypinoresinol and pinoresinol); acetylated hydroxytyrosol; flavonoids (luteolin and apigenin); phenolic acids (p-coumaric acid and vanillic acid); and other secondary substances [14].

The European Parliament approved regulation 1924/2006 [15], which governs nutrition and health claims provided on food products. Regulation No. 1924/2006 gives a definition of “health claims”, which, according to Article 2.5, may be defined as “any claim that states, suggests or implies that a relationship exists between a food category, a food or one of its constituents and health”. Article 2.6 gives a definition of a “reduction of disease risk claim”, intended as “any health claim that states, suggests or implies that the consumption of a food category, a food or one of its constituents significantly reduces a risk factor in the development of a human disease”.

The “use of nutrition and health claims” is authorized by Article 5.1 in labeling, presentation, and advertising of foods placed on the market in the community provided that they are fulfilled under several strict general conditions [15].

In any case, the use of nutrition and health claims, according to the provision of Article 6.1 [15], has to be based on substantiated and generally accepted scientific data. The Regulation [15] states in Article 5.2 that “the use of nutrition and health claims shall only be allowed if the average consumer can be expected to understand the beneficial effects expressed in the claim”.

Currently, four are authorized health claims that can be used for commercial reasons on VOO labels according to EC Regulation No 1924/2006 (article 13, paragraph 1) [15]. These claims are described in EU Regulation No 432/2012 [16], which established a list of permitted health claims made on foods, “other than those referring to the reduction of disease risk and to children’s development and health” (Table 1).

The first and second concepts pertain to the act of substituting saturated fats with unsaturated fats, specifically oleic acid, which is abundantly present in olive oil, in order to “maintain normal blood cholesterol levels”. This health claim can be used for food in which unsaturated fatty acids represent at least 70% of the total fatty acid content and provide more than 20% of the energy of the product.

The third refers to α-linolenic acid and its capacity to “maintain normal blood cholesterol levels”. This health claim could be used in olive oil if it contains at least 0.3 g of α-linolenic acid for 100 g and 100 kcal. It is necessary to inform consumers that 2 g of this acid should be ingested to obtain this beneficial effect.

The fourth concept, which is also relevant to olive oil, pertains to vitamin E and its role in “protection of cells from oxidative stress”. This health claim can be applied to food items that serve as a source of vitamin E, providing a minimum of 1.8 mg per 100 g of the product (equivalent to 15% of the recommended daily intake value of 12 mg for vitamin E).

The fifth health claim focuses on the polyphenols present in olive oil and their role in “protecting blood lipids against oxidative stress”. However, the use of this claim is subject to certain limitations, which are as follows: “The claim may be used only for olive oil which contains at least 5 mg of hydroxytyrosol and its derivatives (e.g., oleuropein complex and tyrosol) per 20 g of olive oil. In order to bear the claim information shall be given to the consumer that the beneficial effect is obtained with a daily intake of 20 g of olive oil”.

Additionally, EC Regulation No 1924/2006 [15] (article 14.1) established a list of permitted health claims made on foods, “referring to the reduction of disease risk and to children’s development and health”. In this group, there is a health claim (Table 1) that could be used in olive oil related again to the practice of replacing saturated fats with unsaturated fats in the diet, and its effect on lowering blood cholesterol levels. This health claim can also be used for food in which unsaturated fatty acids represent at least 70% of total fatty acid content and provide more than 20% of the energy of the product.

Regarding the quantity of the product that can reasonably be expected to be consumed to provide sufficient quantity of the nutrient about which the claim is made, consumers can easily reach the ingestion of 2 g per day to obtain the beneficial effect of maintaining normal blood cholesterol levels (α-linolenic acid), or 20 g daily to gain the protection of blood lipids from oxidative stress (polyphenols). Likewise, vitamin E concentration in EVOO is around 200 mg/kg so, according to the 12 mg recommended daily intake, it would be necessary to consume around 60 g (65 mL) of olive oil daily (about 4 tablespoons) to completely cover the daily reference intake, an amount slightly above the daily intake recommended by the PREDIMED reports (40–50 mL) but easy to reach throughout the day in a Mediterranean diet.

However, the use of this health claim is subjected to a required concentration in olive oil above 250 mg/kg (5 mg/20 g) of phenols (hydroxytyrosol, tyrosol, and complex derivatives), above 3 g/kg of α-linolenic acid (0.3 g/100 g), and above 18 mg/kg (1.8 mg/100 g) of vitamin E (α-tocopherol) throughout the shelf life of olive oil. Therefore, it is extremely important to consider that EVOO is constantly subjected to degradation processes, especially hydrolytic and oxidative reactions that influence its fatty acid, tocopherol, and phenolic composition. External factors, such as light, temperature, and oxygen, as well as other pro-oxidant activators (such as chlorophylls), influence EVOO by increasing the kinetics of oxidative reactions. It has been reported that auto-oxidation phenomena occur naturally in EVOO, even under controlled conditions [18,19,20,21,22].

While EVOOs, especially those with high initial amounts of phenols, have shown a high stability regarding the EU legal quality parameters for the “extra virgin” category (PV, K232, K270) during storage [19,21,22,23], few studies have been done about EVOOs and the evolution of polar phenols, fatty acid, and tocopherol in order to fulfil the health claims all along EVOO shelf life.

The present study aims to assess the impact of lipid oxidation on the nutritional profile of extra virgin olive oil (EVOO) for supporting health claims and to examine the changes in related nutrients during storage in darkness under different usual and accelerated conditions (25, 40, 50, and 60 °C). Fatty acid, tocopherol, and polar phenol compositions were analyzed for both industrially produced EVOOs and EVOOs obtained using the Abencor system. An accelerated method is proposed to determine the end-of-shelf life for EVOOs, not solely based on the loss of the extra virgin commercial category, but especially considering the decline in their nutraceutical capacity and their inability to meet the specified health claims.

## 2. Materials and Methods

### 2.1. Extra Virgin Olive Oil (EVOO) Samples

Five extra virgin olive oils of the Cornicabra variety (III–VII) were graciously provided by industrial oil mills situated in Toledo and Ciudad Real (Castilla-La Mancha, Spain). Two additional extra virgin olive oils (I–II) were derived from Cornicabra olives utilizing the Abencor system (Comercial Abengoa, S.A., Sevilla, Spain) to generate oils with an elevated content of phenolic compounds. All samples underwent filtration with anhydrous Na_2_SO_4_ and were stored in opaque glass bottles devoid of head space at a temperature of 8 °C, shielded from light, until analysis.

### 2.2. Oxidation Experiments

Darkness-stored aliquots of 40 mL (36.6 g) from each EVOO were placed in 125 mL open amber glass bottles (inner diameter: 4.2 cm; air-exposed surface area: 13.85 cm^2^). The bottles were subjected to temperatures of 25, 40, 50, and 60 °C for durations of 93, 41, 34, and 19 weeks, respectively. At determined intervals, one bottle was selected from the incubator for analysis. Two separate batches of samples were employed for each temperature condition under investigation.

### 2.3. Analytical Determinations

All reagents utilized in this study were of analytical, HPLC, or spectroscopic grade and were obtained from Merck (Darmstadt, Germany). All experiments and analytical determinations were conducted in duplicate, or more, to ensure reproducibility and accuracy.

#### 2.3.1. Phenolic Compounds

To a sample of virgin olive oil (2.5 g), 250 μL of a methanol solution containing the internal standard (syringic acid, 15 mg/L) was added. The solvent was evaporated using a rotary evaporator at 35 °C under vacuum. The resulting oil was then dissolved in 6 mL of n-hexane, and a diol-bonded phase cartridge (Supelco Co., Bellefonte, PA, USA) was employed to extract the phenolic fraction. The cartridge was pre-conditioned with 6 mL of methanol and 6 mL of n-hexane. Subsequently, the oil solution was applied to the cartridge, which was then washed with 2 × 3 mL of n-hexane and with 4 mL of n-hexane/ethyl acetate (85:15, *v*/*v*). The phenolic compounds were eluted using 15 mL of methanol, and the solvent was evaporated with a rotary evaporator at 30 °C under vacuum until dryness. The resulting phenolic residue was dissolved in 250 μL of methanol/water (1:1 *v*/*v*).

For HPLC analysis, an Agilent Technologies 1100 series system equipped with an automatic injector, a column oven, and a diode array UV detector was utilized. A Spherisorb S3 ODS2 column (250 mm × 4.6 mm, 5 μm particle size) (Waters Co., Milford, MA, USA) was employed and maintained at a temperature of 30 °C. The injection volume was 20 μL, and the flow rate was set at 1.0 mL/min. The mobile phase consisted of a mixture of water/acetic acid (95:5 *v*/*v*) (solvent A), methanol (solvent B), and acetonitrile (solvent C), with a gradient elution from 95% (A)—2.5% (B)—2.5% (C) to 34% (A)—33% (B)—33% (C) over a period of 50 min. Phenolic compounds were quantified at 280 nm using syringic acid as the internal standard, and the response factors were determined according to Mateos et al. [24].

#### 2.3.2. Tocopherols

The evaluation was performed according to AOCS Method Ce 8–89. An Agilent Technologies HPLC system (1100 series) equipped with a silica gel Lichrosorb Si-60 column (particle size 5 μm, dimensions 250 mm × 4.6 mm i.d.; Sugerlabor, Madrid, Spain) was utilized. The analysis involved a solution of oil in n-hexane. The column was eluted with a mobile phase consisting of n-hexane/2-propanol (98.5:1.5) at a flow rate of 1 mL/min. A fluorescence detector (Thermo-Finnigan FL3000) was employed, with excitation and emission wavelengths set at 290 nm and 330 nm, respectively.

#### 2.3.3. Fatty Acid Composition ([25] and Following Amendments, Corresponding to AOCS Method Ch 2–91)

For the determination of fatty acid composition, the methodology outlined in Mancebo-Campos et al. [20] was employed. To determine the fatty acid composition, the methyl esters were prepared by vigorous shaking of a solution of oil in hexane (0.2 g in 3 mL) with 0.5 mL of 2 N methanolic potassium hydroxide and analyzed by GC with a FID detector. A fused silica column (50 m length × 0.25 mm i.d.) coated with SGL-1000 phase (0.25 μm thickness; Sugerlabor) was used. The carrier gas was helium at a flow rate of 1 mL/min. The injector and detector temperatures were set at 250 °C, and the oven temperature was set at 210 °C. The injection volume was 1 μL. The extent of unsaturated fatty acid loss resulting from oxidation was quantified by comparing the peak areas of each fatty acid to that of palmitic acid. This approach was chosen because saturated fatty acids remain unaffected by autoxidation [26].

### 2.4. Statistical Analysis and Treatment of Experimental Data

The experimental dataset included two separate batches of samples for each temperature condition under investigation. Additionally, duplicate measurements were conducted for each sample at predetermined time intervals. Linear and nonlinear regression analyses were performed using Microsoft Office Excel 2007 for Windows (Microsoft Corporation, Redmond, Washington, DC, USA), and the most suitable equations were selected based on the statistical parameters of the regression analysis (R, p). Statistical analysis, including PCA and MLR, was conducted using SPSS 14 statistical software (SPSS Inc., Chicago, IL, USA). One-way ANOVA was performed using the Tukey test, and statistical significance was considered at *p* < 0.05. The degradation rates of the compounds were calculated based on the slopes of the concentration vs. time experimental curves.

The impact of temperature on reaction rates was assessed using the Arrhenius equation [27]:

(1) $$Ln\;k=Ln\;A-\frac{\mathrm{Ea}}{\mathrm{RT}}$$

where k is the reaction rate constant, R is the molar gas constant (8.31 J K^−1^ mol^−1^), T is the absolute temperature (K), Ea is the activation energy (J mol^−1^), and A is the pre-exponential factor. Given the potential for curvature in the *Ln* k vs. 1/T plot in certain cases, it is reasonable to consider employing a modified equation [28,29]:

(2) $$k={\mathrm{AT}}^{n}\;e^{-\mathrm{Ea}/\mathrm{RT}}$$

where A, n, and Ea are parameters determined using nonlinear fitting (0 < n < 1).

## 3. Results and Discussion

### 3.1. Initial Characteristics of Virgin Olive Oils

As reported in a previous work [30], the EVOO samples analyzed in this study complied with the criteria established by the European Union for the “extra” virgin category. The initial peroxide value (PV) (≤6.5), K232 (≤1.93), and K270 (≤0.16) indicated consistently low levels of oxidation across all seven virgin olive oils investigated. The lipid composition was also similar, as all samples were derived from monovarietal EVOO produced in the same region (Cornicabra cultivar in Castilla-La Mancha, Spain). However, there were slightly, yet statistically significant, variations observed in the content of unsaturated fatty acids (UFAs).

In all presented olive oils, UFAs accounted for more than 70% of the total fatty acid content, specifically between 85% and 87% (Table 2), and thus represented over 20% of their total caloric content, fulfilling the specific conditions for health claims 1 and 2 (Table 1). Likewise, all oils contained more than 0.3 g/100 g of α-linolenic acid, being a source of omega-3 fatty acids (condition for health claim 3, in Table 1), with at least a doubling of that amount in all cases.

Detailed information regarding the phenolic and α-tocopherol contents of the EVOO samples is also provided. All oils contained more than 1.8 mg/100 g (18 mg/kg) of α-tocopherol (vitamin E), specifically between 8 and 13 times more, allowing them to be classified into two groups: very high content (I and III) (more than 200 mg/kg) and high content (II, IV, V, VI, VII) (between 100 and 200 mg/kg).

All oils contained more than 250 mg/kg of hydroxytyrosol, tyrosol, and derivatives (condition to use the EFSA health claim number 5, in Table 1), enabling their classification in two groups: very high content (I, II, and III) (more than 1000 mg/kg) and high content (IV–VII) (between 300 and 1000 mg/kg). Samples III and II had the highest hydroxytyrosol, tyrosol, and derivatives content, followed by sample I. However, sample II showed a higher concentration of dialdehydic forms of oleuropein and ligstroside aglycons (922 mg/kg), while sample III exhibited a higher concentration of aldehydic forms (728 mg/kg), indicating an inverse relationship between both. Samples VII and VI had the lowest hydroxytyrosol, tyrosol, and derivatives content, with the latter displaying the highest levels of free tyrosol (74 mg/kg) and hydroxytyrosol (41 mg/kg), but the lowest levels of secoiridoids complex phenolic compounds (200 mg/kg). Samples IV and V showed similar levels of hydroxytyrosol, tyrosol, and derivatives content, but exhibited contrasting complex/simple phenol ratios. In other words, despite their different complex-simple phenolic profile, all samples contained more than 5 mg of hydroxytyrosol, tyrosol, and derivatives per 20 g of olive oil, certainly from 1.2 to 5 times higher than the requirement set by EFSA for health claim number 5.

### 3.2. Degradation Kinetic of Unsaturated Fatty Acids (UFAs), α-Linolenic Acid (C18:3), α-Tocopherol (TOH) and Hydroxytyrosol, Tyrosol, and Complex Derivatives

Since one of the aims of this work is to propose an accelerated test to determine the end of shelf life for EVOOs based on EFSA health claims, the selected accelerating conditions were temperature and exposure to air to enhance the auto-oxidation process while minimizing the potential confounding effects of photooxidation. It is worth noting that previous research by Krichene et al. [23] indicated that there are no great differences in the degradation of phenolic compounds and α-tocopherol between closed and open bottles.

#### 3.2.1. UFAs and Health Claims Numbers 1 and 2

The evolution of the oxidizing substrate, mainly polyunsaturated fatty acids (PUFA), is depicted in Mancebo-Campos et al. [19]. In all samples and all temperatures (T), the percentage of UFAs in the oils at the end of the experimental period remained above 84%, primarily due to the predominant presence of C18:1 (oleic acid), which remains stable against oxidation to a greater extent than the PUFAs C18:2 and C18:3, which oxidize at a faster rate. Despite their oxidation, the samples studied did not fall at any temperature assayed below the critical threshold of 70 g/100 g required for the inclusion of health claim numbers 1 and 2 on the labeling.

#### 3.2.2. C18:3 and Health Claim Number 3

**Figure 1:**
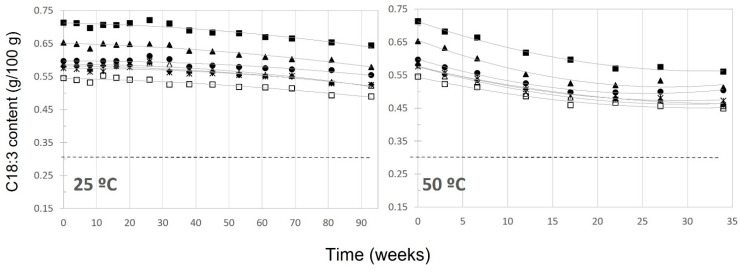
Content of α-linolenic acid (C18:3) during the storage period. Samples: ■, I; ●, II; ▲, III; Δ, IV; ♦, V; ☐, VI; *, VII.

Figure 1 shows the evolution of α-linolenic acid content in all samples at 25 and 50 °C. This polyunsaturated fatty acid decreased almost linearly and at a rate increasing with temperature, presenting a lag phase during the first weeks at 25 °C and a deceleration during the final stages at 40 (Figure S1) and 50 °C. As mentioned before [19], no significant differences were observed in UFA oxidation rates between samples with respect to their initial antioxidant contents; however, at 60 °C the decrease was faster in samples IV–VII (Figure S1). Therefore, in all samples and at all temperatures, the amount of C18.3 in the oil remained above 0.30 g/100 g (condition for the health claim number 3) throughout the entire testing period, except for samples IV and V at 60 °C, which fell below this threshold after 15 and 19 weeks, respectively (Figure S1).

#### 3.2.3. α—Tocopherol and Health Claim Number 4

**Figure 2:**
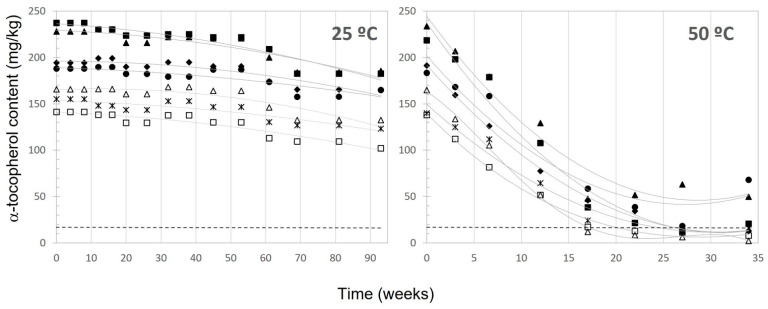
Content of α-tocopherol along the storage period. Samples: ■, I; ●, II; ▲, III; Δ, IV; ♦, V; ☐, VI; *, VII.

The evolution of α-tocopherol and phenolic compound contents in the samples all along the storage periods at 25–60 °C were described in Mancebo-Campos et al. [30]. For α-tocopherol, the decrease was slow and almost linear at 25 (Figure 2) and 40 °C (Figure S2), whereas at 50 (Figure 2) and 60 °C (Figure S2), it dropped rapidly from the beginning, probably due to its oxidation to α-tocopherolquinones [31] and stabilized in all samples when the depletion was significant. At 25 °C and 40 °C, all samples maintained a significantly higher α-tocopherol content throughout the entire testing period, well above the established limit of 18 mg/kg for the health claim number 4. In the study done by Fregapane et al. [32] with commercial oils stored under market conditions, the α-tocopherol lost was between 25 and 30% after 11–12 months, but, despite this, the final concentration exceeded 10 times the limit for the related health claim.

At 50 °C, only samples I, II, and III remained above this limit after 34 weeks, while at 60 °C, nearly all α-tocopherol disappeared after 19 weeks of testing, with only sample II continuing to meet the criteria for the health claim, exhibiting a content of 18.72 mg/kg of α-tocopherol.

#### 3.2.4. Hydroxytyrosol, Tyrosol, and Derivatives and Health Claim Number 5

As also mentioned in Mancebo-Campos et al. [30], in general, hydroxytyrosol and tyrosol increase and their secoiridoids decrease during storage at lower temperatures mainly due to the non-oxidative hydrolysis of the secoiridoids, except in samples with a small content of complex phenols. As the temperature increased, differences between samples concerning their phenolic composition became more evident because of the different rates of hydrolysis of the secoiridoid derivatives, the thermal decomposition of simple and complex phenols, and its action as antioxidants.

**Figure 3:**
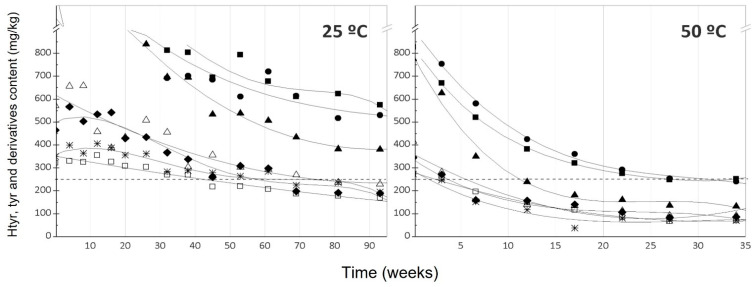
Content of hydroxytyrosol, tyrosol, and derivatives along the storage period. Samples: ■, I; ●, II; ▲, III; Δ, IV; ♦, V; ☐, VI; *, VII.

At 25 °C, only samples I, II, and III, which had the highest content of hydroxytyrosol, tyrosol, and derivatives, maintained a concentration above 250 mg/kg at the end of the trial, while the other samples would not have been able to meet health claim number 5. For samples I and II, the stationary phase of their decline reached above 250 mg/kg at 25 °C (Figure 3) and 40 °C (Figure S3), and close to this concentration at 50 °C (Figure 3) and 60 °C (Figure S3). These samples had an initial content of hydroxytyrosol, tyrosol, and derivatives of 1142 and 1218 mg/kg, respectively, suggesting that with an initial level of hydroxytyrosol, tyrosol, and derivatives above 1100 mg/kg, the health claim could be maintained almost indefinitely. However, the same did not occur with sample III, which had 1276 mg/kg of hydroxytyrosol, tyrosol, and derivatives, as it exceeded the limit of 250 mg/kg at 40 °C, 50 °C, and 60 °C in 24, 13, and 5 weeks, respectively. This could be attributed to its higher o-diphenols/total phenols ratio, as o-diphenols are more susceptible to oxidation and thermal decomposition than tyrosol secoiridoids, as previously reported [19,20,33]. At 50 °C and 60 °C, practically no sample maintained the level of hydroxytyrosol, tyrosol, and derivatives required to retain health claim number 5.

The evolution of α-linolenic acid (Figure 1) followed a pseudo zero-order kinetic, similar to primary oxidation indices previously established [19]. On the contrary, α-tocopherol (Figure 2), the entire concentration-time curve evolution fitted better to a pseudo first-order kinetic, including the stationary phases reached in the last weeks at 50 and 60 °C. The same behavior was observed for the evolution of the sum of hydroxytyrosol, tyrosol, and derivatives content, which showed a better fit to a pseudo first-order kinetic. The experimental rate constants (k) are presented in Table S1.

The temperature dependence of degradation rates for α-linolenic acid, α-tocopherol, and hydroxytyrosol, tyrosol, and derivatives was analyzed using regression analyses based on Ln k vs. 1/T plots. The results indicated that these degradation processes followed the linear Arrhenius model within the temperature range of 25 to 60 °C (Figure S4). The correlation factors (R^2^) obtained were in the range of 0.994 < R^2^ < 0.999 for C18:3, 0.954 < R^2^ < 0.996 for α-tocopherol, and 0.925 < R^2^ < 0.999 for hydroxytyrosol, tyrosol, and derivatives.

### 3.3. Shelf Life of Virgin Olive Oil Related to Health Claims

Based on the investigation of the oxidation parameters and degradation rates of fatty acids within the temperature range of 25 to 60 °C, our research group proposed a parameter called TRUL (Time to Reach the Upper Legal Limit) as a predictor of oxidative stability at 25 °C, based on ASLT at temperatures below 60 °C [19]. Remarkably, when applying the proposed model to accelerated storage temperatures (40, 50, and 60 °C), the predicted TRUL at 25 °C closely matched the experimental TRUL at the corresponding temperature.

Similarly, we explored the possibility of predicting the time it would take for olive oils to lose the feasibility of declaring the health claims described in Table 1, using an accelerated storage test with temperature and exposure to air as the accelerating factors. This time was referred to as TLHC (Time to Lose the Health Claim) and was calculated based on the obtained rate constants (*k*), initial concentrations of α-linolenic acid, α-tocopherol, hydroxytyrosol, tyrosol, and derivatives (C_0_), and the threshold concentrations for each health claim (C). The appropriate rate equations were employed depending on whether the parameter followed a pseudo zero-order (C = C_0_ + *k*∙TLHC) or pseudo first-order kinetics (Ln C = Ln C_0_ + *k*∙TLHC) (see Table S1). The calculated TLHC values and their corresponding experimental values are detailed in Table S2. Most of the calculated values cannot be compared with the experimental ones since the thresholds were not reached during the experimental period (nr). Among those comparable, quite similar were those of α-tocopherol at 50 and 60 °C.

**Figure 4:**
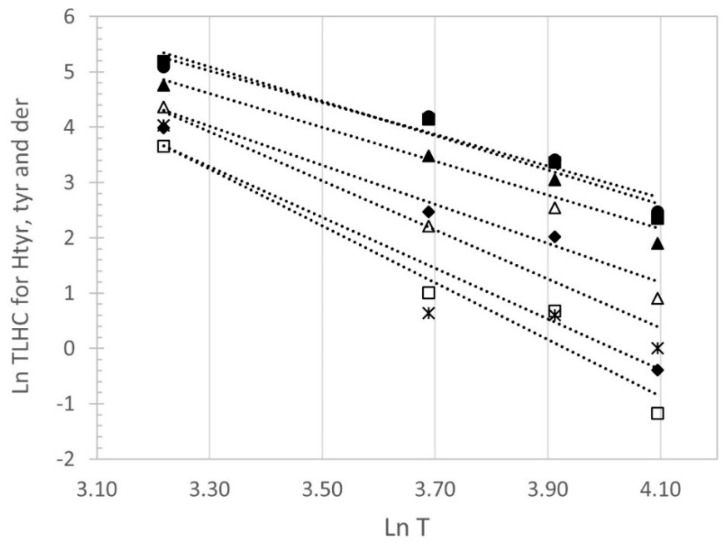
Correlation between TLHC for hydroxytyrosol, tyrosol, and derivatives and temperature (T) TLHC = aT^b^. Samples: ■, I; ●, II; ▲, III; Δ, IV; ♦, V; ☐, VI; *, VII.

These values correlate satisfactorily with temperature (T) using a potential equation, TLHC = aT^b^, as depicted in Figure 4 for hydroxytyrosol, tyrosol, and derivatives and Figure S5 for α-linolenic acid and α-tocopherol (correlation factors shown in Table 3).

Based on the experimental results observed in this study, the feasibility of conducting an accelerated stability test at a temperature below 60 °C was raised to estimate the time it would take for olive oils to lose their ability to declare health-related claims using TLHC for each compound (α-linolenic acid, α-tocopherol or hydroxytyrosol, tyrosol, and derivatives) and the average value of the “b” factor (shown in Table 3).

Table 3 shows the calculated TLHC with the proposed method for all samples and all the parameters by using a mean value for factor “b”.

As an example, the TLHC for hydroxytyrosol, tyrosol, and derivatives at 25 °C, could be predicted from a shorter experimental period at 40 °C:

TLHC_25 °C = a T^b^ b, mean value −3.82 ± 0.89 (see Table 3).

T = temperature of ambient storage (i.e., 25 °C).

T_exp_ = temperature of the accelerated test (i.e., 40 °C).

t_exp_ = time to reach hydroxytyrosol, tyrosol and derivatives = 250 mg/kg at T_exp_ (i.e., 32.6 weeks, for sample III, Table 3).

a = t_exp_/T_exp_ ^−3.82 ± 0.89^ (i.e., a = 32.6/(40^−3.82^)).

TLHC_25 °C = (32.6/(40^−3.82^)) (25^−3.82^) = 197 weeks (for sample III, see Table 3).

In the case of hydroxytyrosol, tyrosol, and derivatives, the comparison between the calculated and actual values is only possible in samples IV, V, VV, and VII, as samples I, II, and III did not reach the limit of 250 mg/kg during the experimental period at 25 °C. Only the calculated values in samples IV and V from an accelerated test at 40 °C show reasonable similarity to the experimental TLHC in the studied EVOO samples. For the remaining samples and temperatures, the calculated TLHC values were very different from the experimental values at 25 °C. For the compounds α-linolenic acid and α-tocopherol, the TLHC at 25 °C could be estimated from tests at 40–60 °C; however, it has not been possible in this case to verify their correspondence with the actual TLHC at 25 °C, as the calculated values have always been higher than the 93 weeks of storage conducted.

Therefore, the attempt to conduct a single test at a sole temperature between 40 and 60 °C and using the potential equation TLHC = aT^b^ to calculate the TLHC at 25 °C has proven unfeasible due to the low similarity observed between the calculated and experimental values. In the case of α-linolenic acid, its stability, even at temperatures of 60 °C, requires a long testing time (around 30 weeks) to reach the limit of 0.3 g/100 g established for the health claim, and establish a TLHC at 25 °C above 400 weeks, a time that greatly exceeds the shelf life based on the commercial category of extra virgin oil. Similarly, for α-tocopherol, it would be necessary to conduct this test at 40 °C or 50 °C for more than 60 or 20 weeks, respectively, to conclude that the TLHC at 25 °C of the oil would exceed 300 weeks. Lastly, for hydroxytyrosol, tyrosol, and derivatives, the impossibility lies in using a standard “b” parameter, an average of the samples in this test, to calculate the TLHC. The variability (SD = 0.89) of this parameter seems to be high enough that the calculated value and the actual value differ considerably in many samples.

## 4. Conclusions

With the aim of establishing an accelerated method to determine the shelf life of extra virgin olive oil, this research group conducted a study on the behavior of normalized oxidation parameters and the lipid matrix, resulting in a formula to predict the time required to reach shelf life considering the loss of the “extra virgin” quality grade. In the present study, a similar approach was attempted but with the consideration of potential health claims that can be labeled on extra virgin olive oils as a quality attribute that must be maintained throughout the entire shelf life.

It was possible to calculate the degradation rate constant (*k*) of the a-linolenic acid, a-tocopherol, and hydroxytyrosol, tyrosol, and derivatives in an olive oil at 25 °C after carrying out an accelerated test at three higher temperatures with the same olive oil. With these rate constants, the initial concentrations, and the threshold concentrations could be calculated the shelf life based on health claims at 25 °C, by means of the equations C = C_0_ + kt or Ln C = Ln C_0_ + kt.

The evolution of the phenolic compound content, including hydroxytyrosol, tyrosol, and their secoiridoid derivatives, in relation to their initial content in the sample and the testing temperature, is a matter of consideration, since this leads to varying rates of hydrolysis for complex hydroxytyrosol and tyrosol secoiridoids, as well as degradation of simple phenols, making it difficult to predict in advance.

## Figures and Tables

**Table 1:** EFSA health claims with potential use in virgin olive oils.

Nutrient or Substance in the Food or Food Category	Declaration	Conditions of Use
Monounsaturated and/or polyunsaturated fatty acids	(1) Replacing saturated fats with unsaturated fats in the diet contributes to the maintenance of normal blood cholesterol levels [MUFA and PUFA are unsaturated fats]	The claim may be used only for food, which is high in unsaturated fatty acids, as referred to in the claim HIGH UNSATURATED FAT as listed in the Annex to Regulation (EC) No 1924/2006 [15].
	(2) Replacing saturated fats with unsaturated fats in the diet has been shown to lower/reduce blood cholesterol. High cholesterol is a risk factor in the development of coronary heart disease	
Alpha-linolenic acid (ALA)	(3) ALA contributes to the maintenance of normal blood cholesterol levels	The claim may be used only for food which is at least a source of ALA as referred to in the claim SOURCE OF OMEGA-3 FATTY ACIDS as listed in the Annex to Regulation (EC) No 1924/2006 [15]. Information shall be given to the consumer that the beneficial effect is obtained with a daily intake of 2 g of ALA.
Vitamin E	(4) Vitamin E contributes to the protection of cells from oxidative stress	The claim may be used only for food which is at least a source of vitamin E as referred to in the claim SOURCE OF [NAME OF VITAMIN/S] AND/OR [NAME OF MINERAL/S] as listed in the Annex to Regulation (EC) No 1924/2006 [15].
Olive oil polyphenols	(5) Olive oil polyphenols contribute to the protection of blood lipids from oxidative stress	The claim may be used only for olive oil, which contains at least 5 mg of hydroxytyrosol and its derivatives (e.g., oleuropein complex and tyrosol) per 20 g of olive oil. In order to bear the claim, information shall be given to the consumer that the beneficial effect is obtained with a daily intake of 20 g of olive oil.

(1), (3), (4) and (5) Health claims other than those referring to the reduction of disease risk and to children’s development and health [16]. (2) Health claim referring to the reduction of disease risk [17].

**Table 2:** Initial Composition of Olive Oil Samples.

	EVOO Samples						
	*I*	*II*	*III*	*IV*	*V*	*VI*	*VII*
C18:3 ^(1)^	0.71 ± 0.00 ^f^	0.59 ± 0.00 ^d^	0.65 ± 0.00 ^e^	0.58 ± 0.00 ^c^	0.58 ± 0.00 ^c^	0.54 ± 0.00 ^a^	0.57 ± 0.00 ^b^
UFAs ^(1)^	84.8 ± 0.2 ^a^	85.7 ± 0.0 ^d^	85.5 ± 0.0 ^c^	85.2 ± 0.0 ^b^	86.8 ± 0.0 ^f^	85.6 ± 0.0 ^c^	86.6 ± 0.0 ^e^
Htyr, Tyr and derivatives ^(2)^	1142 ± 0 ^d^	1218 ± 145 ^d,e^	1276 ± 10 ^e^	468 ± 19 ^c^	443 ± 9 ^b,c^	314 ± 5 ^a^	335 ± 1 ^a,b^
mg Htyr, Tyr and derivatives/20 g of oil	22.8 ± 0.0 ^d^	24.4 ± 2.9 ^d,e^	25.5 ± 0.2 ^e^	9.4 ± 0.4 ^c^	8.9 ± 0.2 ^b,c^	6.3 ± 0.1 ^a^	6.7 ± 0.0 ^a,b^
α-Tocopherol ^(2)^ (vit. E)	238.0 ± 4.1 ^f^	188.4 ± 2.2 ^d^	227.2 ± 6.5 ^e^	164.7 ± 2.2 ^c^	193.8 ± 0.0 ^d^	141.1 ± 2.2 ^a^	155.1 ± 0.0 ^b^

Mean values with different letters in the same row are statistically different (*p* ≤ 0.05). ^1^ Expressed as g/100 g; ^2^ Expressed as mg/kg.

**Table 3:** Comparison between the experimental and predicted TLHC (weeks) for α-linolenic, and hydroxytyrosol, tyrosol, and its derivatives by means of the proposed method based on a potential equation (TLHC = aT^b^).

		TLHC (Weeks)						
	Samples	Real at 25 °C	Exp at 40 °C	Calc at 25 °C	Exp at 50 °C	Calc at 25 °C	Exp at 60 °C	Calc at 25 °C
0.9719 < * R^2^ < 0.9987; = −3.82 ± 0.89								
Htyr, tyr and derivatives = 250 mg/kg	I	nr	63.0	379	28.7	406	10.5	296
	II	nr	66.3	399	30.1	425	11.8	336
	III	nr	32.6	197	21.1	298	6.7	190
	IV	69–81	9.2	55	12.7	179	2.5	70
	V	61–69	11.8	71	7.5	106	0.7	19
	VI	38–45	2.7	16	2.0	28	0.3	9
	VII	61–69	1.9	11	1.8	26	0	0
0.9922 < * R^2^ < 0.9998/b = −3.15 ± 0.18								
	I	nr	135.4	595.0	64.7	574.4	32.5	512.9
C18:3 = 0.30 g/100 g	II	nr	145.5	639.3	62.6	555.7	33.5	528.5
	III	nr	113.3	498.0	54.0	479.8	28.4	448.0
	IV	nr	110.8	487.0	59.7	529.6	26.8	422.4
	V	nr	125.2	550.2	64.6	573.5	27.7	436.3
	VI	nr	137.0	602.1	63.9	566.8	30.7	484.4
	VII	>nr	>131.2	>576.6	>59.3	526.0	31.9	502.7
0.9693 < * R^2^ < 0.9992/b = −5.13 ± 0.34								
α-tocopherol = 18 mg/kg	I	nr	62.6	483.7	18.5	376.7	9.5	426.3
	II	nr	119.1	919.9	24.9	508.2	10.7	482.2
	III	nr	83.2	642.5	19.5	397.3	10.2	460.4
	IV	nr	61.5	475.4	16.5	337.4	7.6	343.7
	V	nr	82.2	635.2	20.9	427.2	8.7	392.5
	VI	nr	86.7	670.1	17.1	348.0	8.0	362.8
	VII	nr	96.7	747.4	18.1	369.6	8.1	365.7

nr = the limit value was not reached during the experimental period; Exp = experimental weeks. * R^2^ from TLHC vs. T, fitted as TLHC = aT^b^.

## Data Availability

Data are available in the manuscript and supplementary file.

## Supplementary Materials

The following supporting information can be downloaded at: https://www.mdpi.com/article/10.3390/antiox12081563/s1, Table S1: Degradation rate constants (k) for α-linolenic acid (C18:3), α-tocopherol, and hydroxytyrosol, tyrosol, and its derivatives at the experimental temperatures studied; Table S2: Calculated Time (weeks) required to reach the limits for Declining the Health Claims (TLHC, weeks) for α-linolenic acid (C18:3), α-tocopherol, and hydroxytyrosol, tyrosol, and its derivatives as compared with the experimental ones; Figure S1: Content of α-linolenic acid (C18:3) along the storage period at 40 and 60 °C; Figure S2: Content of α-tocopherol along the storage period at 40 and 60 °C; Figure S3: Content of hydroxytyrosol, tyrosol, and derivatives along the storage period at 40 and 60 °C; Figure S4. Arrhenius plot: Effect of temperature (T) on oxidation rate constants (k); Figure S5: Correlation between TLHC for α-linolenic acid (C18:3) and α-tocopherol (TOH) and temperature (T).
