# Supplementary material for: EFSA Health Claims-Based Virgin Olive Oil Shelf-Life
Source: Antioxidants (Basel). 2023 Aug 4;12(8):1563. doi: 10.3390/antiox12081563 (PMC10452016; doi:10.3390/antiox12081563)
Supplement: Supplementary file 1 [file antioxidants-12-01563-s001.zip › antioxidants-2535038-supplementary.pdf]

Table S1. Degradation rate constants (*k*) for α-linolenic acid (C18:3), α-tocopherol, and hydroxytyrosol, tyrosol and its derivatives at the experimental temperatures studied.

	Sample	**25 ºC**		**40 ºC**		**50 ºC**		**60 ºC**	
		***k*/10^-3^**	***R^2^**	***k*/10^-3^**	***R^2^**	***k*/10^-3^**	***R^2^**	***k*/10^-3^**	***R^2^**
**C18:3** [1] *k*/% weeks^{-1}	***I***	-0.76	0.853	-3.05	0.916	-6.38	0.985	-12.69	0.986
	II	-0.42	0.787	-2.04	0.892	-4.74	0.955	-8.85	0.989
	III	-0.75	0.896	-3.11	0.909	-6.52	0.957	-12.4	0.983
	IV	-0.69	0.858	-2.59	0.913	-4.81	0.893	-10.71	0.990
	V	-0.64	0.842	-2.24	0.881	-4.34	0.954	-10.13	0.977
	VI	-0.60	0.876	-1.79	0.859	-3.84	0.921	-7.98	0.970
	VII	-0.54	0.696	-2.10	0.931	-4.65	0.980	-8.64	0.971
		***k* /10^-2^**	***R^2^**	***k* /10^-2^**	***R^2^**	***k* /10^-2^**	***R^2^**	***k* /10^-2^**	***R^2^**
**α-tocopherol** [2] *k*/weeks^{-1}	***I***	-0.30	0.849	-2.20	0.968	-9.12	0.884	-18.65	0.971
	II	-0.19	0.703	-0.95	0.918	-5.41	0.637	-12.57	0.973
	III	-0.26	0.798	-1.42	0.919	-5.16	0.803	-20.17	0.968
	IV	-0.27	0.714	-2.02	0.954	-13.34	0.973	-30.59	0.955
	V	-0.21	0.776	-1.46	0.958	-8.76	0.971	-28.51	0.944
	VI	-0.35	0.850	-1.21	0.932	-9.25	0.949	-26.62	0.960
	VII	-0.25	0.776	-1.14	0.970	-7.81	0.906	-25.76	0.949
		***k* /10^-2^**	***R^2^**	***k* /10^-2^**	***R^2^**	***k* /10^-2^**	***R^2^**	***k* /10^-2^**	***R^2^**
**Htyr, tyr and derivatives** [2] *k*/weeks^{-1}	***I***	-0.846	0.866	-1.88	0.840	-4.17	0.964	-10.95	0.980
	II	-0.974	0.845	-1.49	0.844	-4.29	0.974	-8.80	0.972
	III	-1.39	0.909	-2.89	0.772	-5.37	0.911	-14.85	0.933
	IV	-1.05	0.804	-2.23	0.789	-4.02	0.825	-13.05	0.958
	V	-1.15	0.838	-2.33	0.896	-4.25	0.926	-11.20	0.994
	VI	-0.656	0.852	-2.08	0.893	-4.53	0.995	-10.17	0.990
	VII	-0.587	0.738	-2.05	0.849	-4.76	0.940	-10.94	0.985

[1] Pseudo zero-order kinetics: $C = C_0 + kt$ *P<0.05

[2] Pseudo first-order kinetics: $\ln C = \ln C_0 + kt$

Table S2. Calculated Time (weeks) required to reach the limits for Declining the Health Claims (TLHC, weeks) for α-linolenic acid (C18:3), α-tocopherol, and hydroxytyrosol, tyrosol and its derivatives as compared with the experimental ones

	Sample	**25 ºC**		**40 ºC**		**50 ºC**		**60 ºC**	
		**TLHC**	**Exp**	**TLHC**	**Exp**	**TLHC**	**Exp**	**TLHC**	**Exp**
**C18:3**	***I***	543.2	nr	135.4	nr	64.7	nr	32.5	nr
= 0.30 g/100 g	***II***	706.5	nr	145.5	nr	62.6	nr	33.5	nr
	III	469.9	nr	113.3	nr	54.0	nr	28.4	nr
	IV	415.9	nr	110.8	nr	59.7	nr	26.8	12.5
	V	438.1	nr	125.2	nr	64.6	nr	27.7	19.0
	VI	408.7	nr	137.0	nr	63.9	nr	30.7	17.5
	VII	510.2	nr	131.2	nr	59.3	nr	31.9	nr
		**TLHC**	**Exp**	**TLHC**	**Exp**	**TLHC**	**Exp**	**TLHC**	**Exp**
**α-tocopherol**	***I***	859.4	nr	112.8	nr	27.4	24	13.8	13
= 18 mg/kg	***II***	1234.4	nr	237.2	nr	42.9	27	18.9	19
	III	976.3	nr	172.9	nr	49.7	nr	12.8	16
	IV	822.3	nr	105.1	nr	16.6	16	7.3	7
	V	1132.5	nr	155.5	nr	27.0	26	8.3	9
	VI	588.4	nr	156.8	nr	22.0	16	7.7	8.5
	VII	861.1	nr	176.5	nr	26.2	23	8.2	9
		**TLHC**	**Exp**	**TLHC**	**Exp**	**TLHC**	**Exp**	**TLHC**	**Exp**
**Htyr, tyr and derivatives**	***I***	179.6	nr	63.0	nr	28.7	27	10.5	16
= 250 mg/kg	***II***	162.6	nr	66.3	nr	30.1	30	11.8	nr
	III	117.2	nr	32.6	25	21.1	11	6.7	3
	IV	78.8	69-81	9.2	10	12.7	5	2.5	1
	V	53.9	61-69	11.8	11	7.5	4	0.7	1
	VI	38.6	38-45	2.7	9	2.0	3	0.3	0.5
	VII	56.7	61-69	1.9	8	1.8	3	0.0	0.5

nr =the limit value was not reached during the experimental period; Exp = approximate experimental weeks

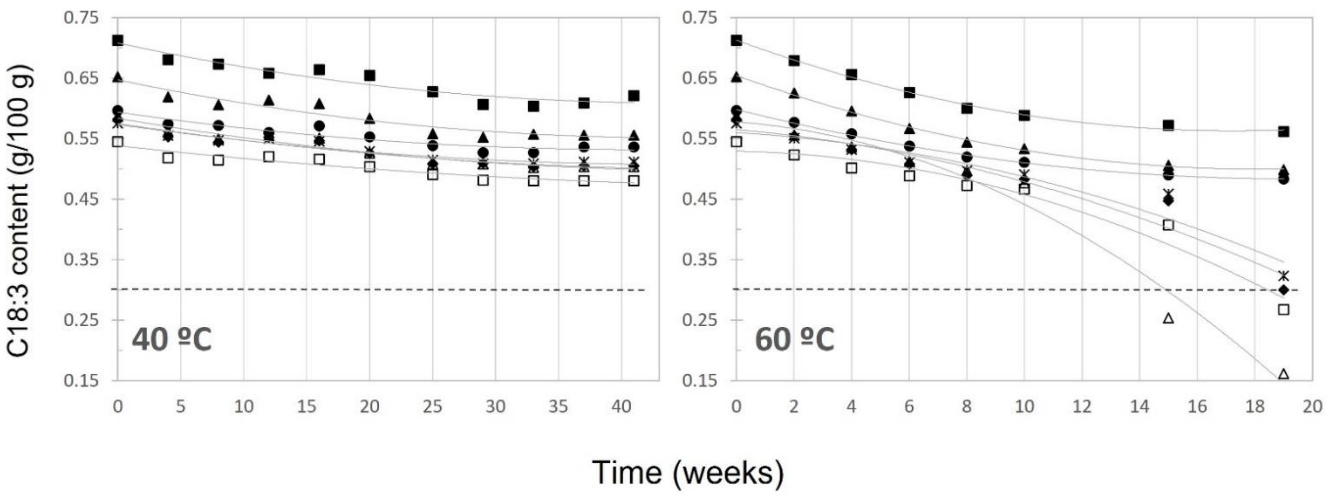

Figure S1. Content of α-linolenic acid (C18:3) along the storage period at 40 and 60 ºC
Samples: ■, I; •, II; ▲, III; Δ, IV; ♦, V; □, VI; *, VII

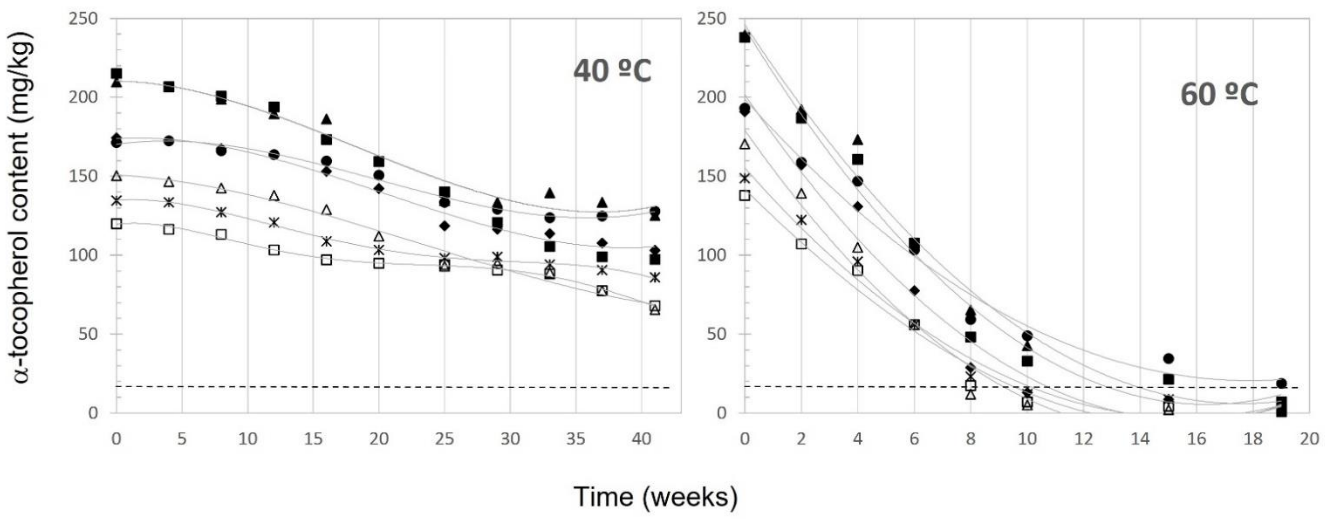

Figure S2. Content of α-tocopherol along the storage period at 40 and 60 ºC
Samples: ■, I; •, II; ▲, III; Δ, IV; ♦, V; □, VI; *, VII

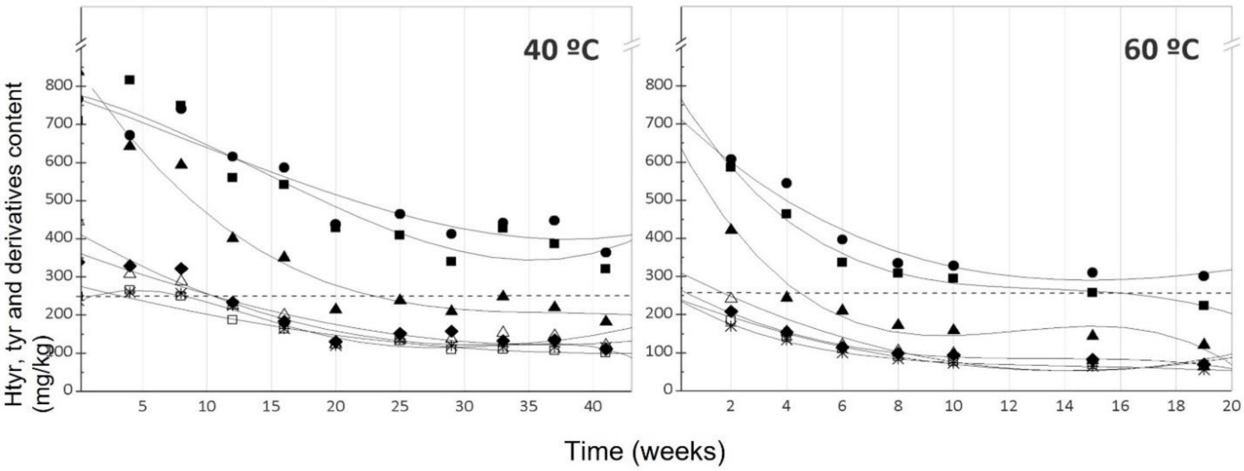

Figure S3. Content of hydroxytyrosol, tyrosol and derivatives along the storage period at 40 and 60 ºC

Samples: ■, I; •, II; ▲, III; Δ, IV; ♦, V; □, VI; *, VII

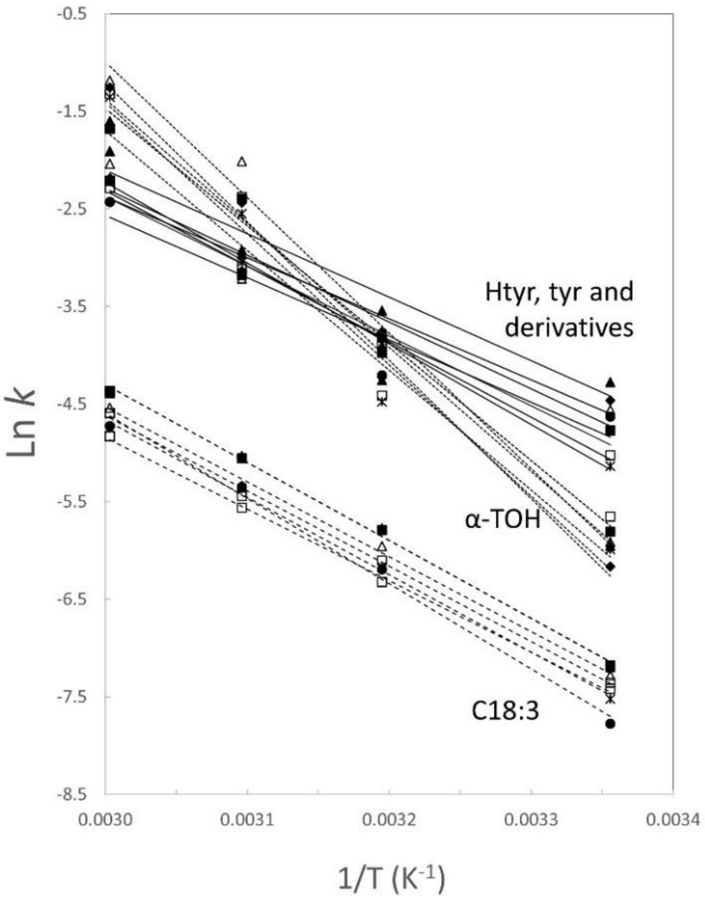

Figure S4. Arrhenius plot: Effect of temperature (T) on oxidation rate constants (k) $\boldsymbol{Lnk = Ln\ A\ - \frac{Ea}{RT}}$

Samples: ■, I; •, II; ▲, III; Δ, IV; ♦, V; □, VI; *, VII

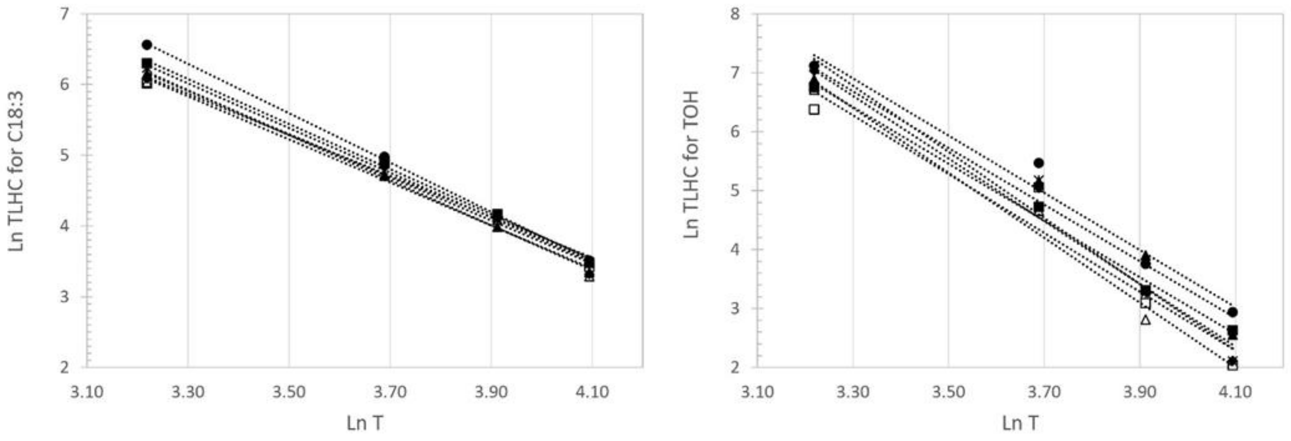

Figure S5. Correlation between TLHC for α-linolenic acid (C18:3) and α-tocopherol (TOH) and temperature (T)

TDHC = aT^b Samples: ■, I; •, II; ▲, III; Δ, IV; ♦, V; □, VI; *, VII
